# Molecular characterisation of rare loss-of-function *NPAS3* and *NPAS4* variants identified in individuals with neurodevelopmental disorders

**DOI:** 10.1038/s41598-021-86041-4

**Published:** 2021-03-23

**Authors:** Joseph J. Rossi, Jill A. Rosenfeld, Katie M. Chan, Haley Streff, Victoria Nankivell, Daniel J. Peet, Murray L. Whitelaw, David C. Bersten

**Affiliations:** 1 Department of Molecular and Biomedical Science, University of Adelaide, Adelaide, 5005 Australia; 2 Department of Molecular and Human Genetics, Baylor College of Medicine, Houston, TX 77030 USA; 3 Department of Clinical Genomics, Baylor Genetics Laboratory, Houston, TX 77030 USA

**Keywords:** Disease genetics, Mutation, Neurodevelopmental disorders

## Abstract

Aberrations in the excitatory/inhibitory balance within the brain have been associated with both intellectual disability (ID) and schizophrenia (SZ). The bHLH-PAS transcription factors NPAS3 and NPAS4 have been implicated in controlling the excitatory/inhibitory balance, and targeted disruption of either gene in mice results in a phenotype resembling ID and SZ. However, there are few human variants in *NPAS3* and none in *NPAS4* that have been associated with schizophrenia or neurodevelopmental disorders. From a clinical exome sequencing database we identified three *NPAS3* variants and four *NPAS4* variants that could potentially disrupt protein function in individuals with either developmental delay or ID. The transcriptional activity of the variants when partnered with either ARNT or ARNT2 was assessed by reporter gene activity and it was found that variants which truncated the NPAS3/4 protein resulted in a complete loss of transcriptional activity. The ability of loss-of-function variants to heterodimerise with neuronally enriched partner protein ARNT2 was then determined by co-immunoprecipitation experiments. It was determined that the mechanism for the observed loss of function was the inability of the truncated NPAS3/4 protein to heterodimerise with ARNT2. This further establishes *NPAS3* and *NPAS4* as candidate neurodevelopmental disorder genes.

## Introduction

Individuals with schizophrenia (SZ; OMIM: 181500) present with positive symptoms described as a lost sense of reality (for example, delusions and hallucinations), and negative symptoms including general apathy and social withdrawal^1,2^. In addition, individuals with SZ often present with reduced cognitive ability and are commonly diagnosed with intellectual disability (ID)^3^, which is characterised by reduced intelligence (IQ < 70) and adaptive behaviour diagnosed before 18 years of age^4^. A strong genetic component has been attributed independently to both ID and SZ, with a group difference heritability of 0.46^5^ and heritability of 0.5–0.87^6^ respectively. For both mild ID and SZ, the underlying genetic cause often appears to be genes of small effect and results in a polygenic inheritance pattern^1,4,5^. Combined with the observation that family members of an individual with SZ may present with some features of SZ (including ID)^6,7^, it seems likely that ID and SZ share common genetic components^1,2,8^. This agrees with the observation that both SZ and ID can present with interneuron defects that disrupt the excitatory/inhibitory balance^9^. Both Neuronal Per-Arnt-Sim 3 (NPAS3) and NPAS4 are known to influence the excitatory/inhibitory balance and are associated with ID and SZ in mouse knockout models, however, directed research linking these factors to human neurodevelopmental disorders is required to support the proposal that they contribute to ID and/or SZ.

NPAS3 is expressed in regions of developing interneurons and throughout the postnatal brain, including cortical and hippocampal interneurons^10,11^. During development NPAS3 has an apparent role in neurogenesis, as it was observed that *Npas3*^−/−^ mice exhibit decreased numbers of cortical interneurons^11^. A decrease in the number of cortical interneurons could lead to a disruption of the excitatory/inhibitory balance, which has been linked to SZ and ID^9^. Hyperexcitability in hippocampal neurons was observed in *Npas3*^−/−^ mice^12^, and it has also been observed that *Npas3*^−/−^ mice have learning issues linked to the hippocampus^13^. Additionally, *Npas3*^−/−^ mice showed deficits in adult neurogenesis within the dentate gyrus of the hippocampus^14^ which was later established to result from decreased survival of neural progenitors^12^. A reduction in adult neurogenesis is also seen in individuals with SZ^15^. Individuals with SZ also perform worse in the pre-pulse inhibition test^16^, a phenotype which is present in the *Npas3*^−/−^ mice^13^. Finally, the *Npas3*^−/−^ mice present with decreased reelin expression^10,17^, notable because a loss of reelin in mice and humans is associated with both SZ- and reduced cognitive ability^18,19^.

The first identified human *NPAS3* variant was a translocation that occurs early in the *NPAS3* gene in a mother and daughter with SZ and variable ID^20,21^. The same translocation was also identified in the son who presented with cognitive deficits^20^. In agreement with this early observation, two other variants that are predicted to cause frameshifts in *NPAS3* have recently been identified in individuals with SZ^22,23^. Only one characterised *NPAS3* single nucleotide polymorphism (SNP; V304I) with a weak reduction in reporter gene activity, has been shown to co-segregate with SZ in a small family^24,25^. Another study has reported an association between *NPAS3* variants and SZ, however, no functional assays were performed^26^. Finally, an *NPAS3* variant has also been associated with atypical Sotos syndrome (predominant phenotype of developmental delay/low IQ without overgrowth or dysmorphic features), where a deletion that removed the first exon of *NPAS3* was identified in the proband^27^. However, the same *NPAS3* allele was also identified in the healthy mother^27^. Altogether, these observations suggest a likely role for NPAS3 in ID and SZ. However, to date there are only two studies that evaluate the functional consequences of 3 *NPAS3* variants^24,28^ and as such there is a lack of characterised *NPAS3* variants that are associated with human pathology.

*Npas4* is expressed primarily in the adult mouse brain within the anterior olfactory nucleus, hippocampus and several cortical structures^29^. The expression of NPAS4 is rapidly upregulated upon depolarisation of both excitatory and inhibitory neurons^30,31^, and while the early gene response is similar between neuron types, the NPAS4 controlled late gene response varies in excitatory and inhibitory neurons^31^. In excitatory neurons, unique NPAS4 regulated genes such as *Bdnf* (Brain Derived Neurotrophic Factor) are important for the formation of inhibitory synapses onto excitatory neurons^30,31^. In contrast, within interneurons NPAS4 controls a different subset of genes that regulate the formation of excitatory inputs onto the interneurons^31^. NPAS4 has different roles in excitatory and inhibitory neurons and it is able to regulate levels of excitation and inhibition^30,31^. As the excitatory/inhibitory balance is often dysfunctional in ID and SZ^9^, it is possible that loss-of-function *NPAS4* variants could contribute to these pathologies. In agreement with this, *Npas4* null mice have deficits in short- and long-term memory, working memory and cognitive flexibility^32,33^. This may be partially explained by the observed decrease in *Bdnf* in the absence of *Npas4*^30^, as a polymorphism in *BDNF* is associated with memory deficits in both mouse models and human subjects^34,35^. Finally, the *Npas4*^−/−^ mice also possess a behavioural phenotype reminiscent of SZ^32^.

The involvement of *NPAS4* in human pathology is not well characterised. Only one deletion (~ 1 Mb) that encompasses the entirety of *NPAS4* has been reported in an individual with ID^36^. While the loss of *NPAS4* is a viable candidate for the observed ID, a definitive conclusion is not possible. Additionally, a GWAS study found that *NPAS4* variants were in linkage disequilibrium with a SNP strongly associated with bipolar disorder^37^ which has been shown to share genetic risk factors with SZ^38^. Previously, variants of *NPAS4* identified in a public database have been characterised^39^, but patient phenotype information was unavailable and no genotype–phenotype correlations could be made. Therefore, more work is needed to identify *NPAS4* variants that are linked to SZ or neurodevelopmental disorders.

Despite the importance of NPAS3 and NPAS4 in neuronal function, there is currently a lack of characterised human variants associated with ID and SZ. Therefore, we identified individuals from a clinical exome sequencing database who possessed a *NPAS3* or *NPAS4* variant that may reduce transcriptional activity and a phenotype that included either ID or developmental delay. We found that truncating mutations in both NPAS3 and NPAS4 that disrupt PAS A result in a complete loss of transcriptional activity due to an inability to heterodimerise with partner protein ARNT2, as well as reduced protein expression. We also determined that disruption of the NPAS3 PAS B domain is sufficient to inhibit heterodimerisation and transcriptional activity. Therefore, we have identified the first variant in *NPAS4* identified in an individual with developmental delay, as well as the third *NPAS3* variant in an individual with developmental delay/ID, further establishing *NPAS3* and *NPAS4* as candidate neurodevelopmental disorder genes.

## Results

### *NPAS3* and *NPAS4* variants identified in exome sequencing database

All basic-Helix-Loop-Helix-PAS (bHLH-PAS) transcription factors have a conserved domain architecture, comprised of an N-terminal bHLH domain that is involved in DNA binding as well as dimerisation, followed by two PAS repeats, PAS A and PAS B, that are involved in dimerisation and have the potential to bind ligands or contact DNA^40,41^. Following the PAS repeats are C-termini that often lack well characterised domains and may either activate or repress transcription^42–45^. There are two classes of bHLH-PAS transcription factors, where class I factors such as NPAS3 and NPAS4 must heterodimerise with a class II factor such as ARNT (ARyl-hydrocarbon Nuclear Translocator) or ARNT2^40^. It has been repeatedly demonstrated that in the absence of a class II factor, class I factors are unable to bind DNA and influence transcription^46–48^. We previously reported that loss-of-function missense variants in the class I factor Single Minded 1 (SIM1) that are associated with obesity tend to disrupt dimerisation with ARNT2 and cluster around the PAS B domain but can also occur in the bHLH domain^43^. In addition, a missense variant within the PAS A domain of NPAS4 was shown to cause a loss of function due to an inability to heterodimerise with ARNT2^39^. We reasoned that selecting variants with significant amino acid changes that occur close to the functional domains in the N-terminus and near previously identified loss-of-function variants, would increase the likelihood of identifying variants that cause a reduction in NPAS3/NPAS4 transcriptional activity.

**Figure 1:**
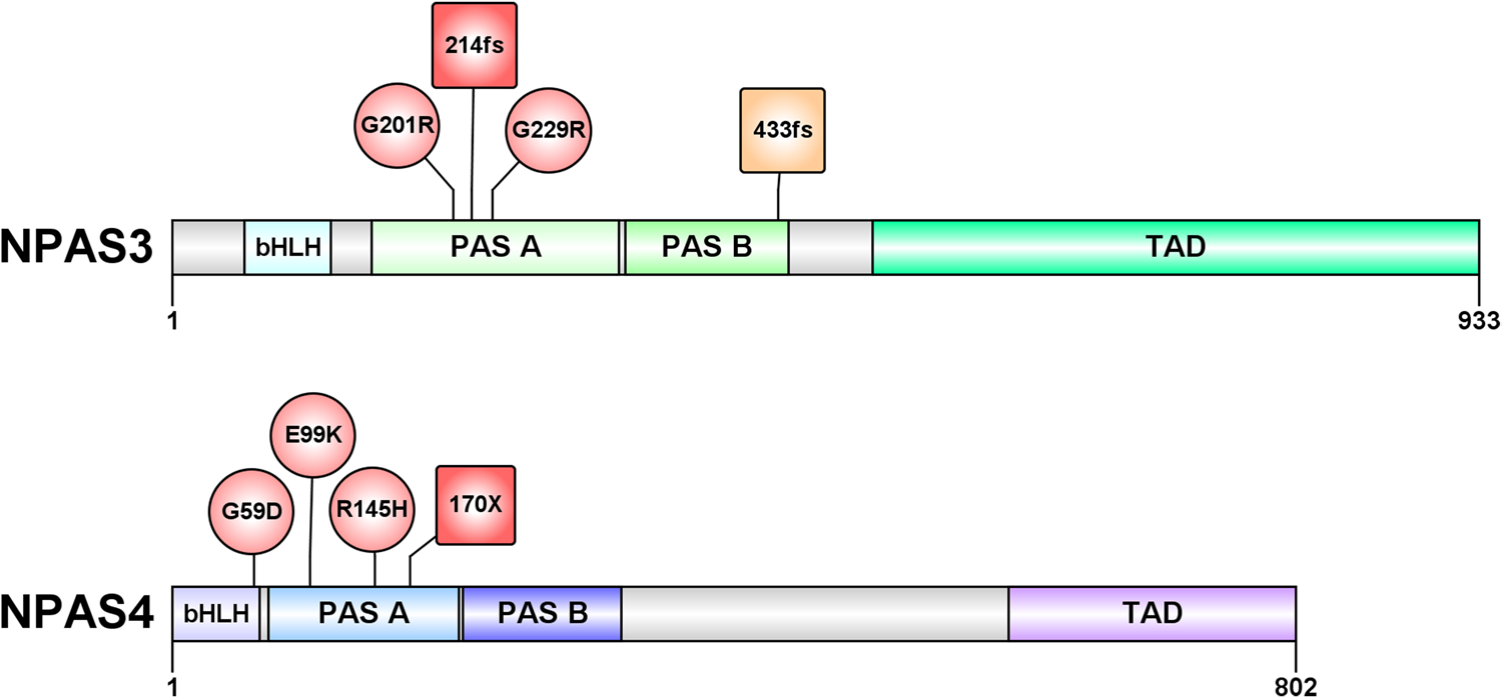
*NPAS3* and *NPAS4* variants characterised. Variants that were identified through exome sequencing in patients with intellectual disability or developmental delay are indicated in red. A mutation that was characterised but not found in a patient (NPAS3 433fs) is indicated in orange. Missense variants are labelled by circular boxes and truncating mutations are in square boxes. N-terminal domain boundaries were determined based on alignments performed for the NPAS1/NPAS3 crystal structure^51^. *bHLH*—basic Helix–Loop–Helix, *PAS*—Per Arnt Sim, *TAD*—trans-activation domain, *NPAS3*—neuronal PAS 3, *NPAS4*—neuronal PAS 4, *fs*—frameshift variant, *X*—nonsense variant.

We selected three variants in NPAS3 from the Baylor Genetics clinical exome sequencing database which cluster around the PAS A domain (Fig. 1 and Supplementary Fig. S1). Two variants identified in NPAS3, G201R and G229R, were deemed to impact protein function by in-silico prediction software (Table 1). These variants are present at a low frequency (< 0.004%) in the Genome Aggregation Database (gnomAD^49^) “non-neuro” population (Table 1), which represents approximately 115,000 genomes and exomes from individuals that are not related and were not diagnosed with a neurological illness. The presence of these alleles at a low frequency in the population is consistent with the alleles potentially being deleterious, as a deleterious allele is unlikely to be common. We also selected a frameshift variant at position 214 of the NPAS3 protein (214fs) that is absent from gnomAD (Table 1). The frameshift causes the introduction of 12 novel amino acids and a premature termination codon (PTC) which results in a significant part of NPAS3 being lost, including the C-terminal transactivation domain. Therefore, NPAS3 214fs likely results in a non-functional protein.

**Table 1:** Variant frequency and in silico predictions.

	AA change (Nt change)	gnomAD database frequency (v2.1.1 “non-neuro”)	In silico predictions		
			Polyphen2 HumDiv^†^^68^	PMut 2017^69^	SNAP2^70^
NPAS3*	G201R (c.601 G>A)	0.00003370	Probably damaging	Disease	Effect
	G229R (c.685G>A)	0.00003550	Probably damaging	Disease	Effect
	214fs (c.641dupT)	Absent	N/A	N/A	N/A
NPAS4**	G59D (c.176G>A)	0.00002890	Possibly damaging	Neutral	Neutral
	E99K (c.295G>A)	0.000009611	Benign	Disease	Effect
	R145H (c.434G>A)	0.000009625	Probably damaging	Disease	Effect
	170X (c.508C>T)	Absent	N/A	N/A	N/A

*NPAS3 refseq: NM_001164749.2; **NPAS4 ref seq: NM_178864.4; ^†^Probably Damaging > Possibly Damaging > Benign.

*AA*—amino acid, *N/A*—not applicable, *Nt*—nucleotide.

We selected four variants in NPAS4 from the Baylor Genetics clinical exome sequencing database which occur in the N-terminal half of the protein (Fig. 1). Three missense substitutions were identified in NPAS4, G59D, E99K and R145H, which occur at highly conserved residues across neuronal bHLH-PAS proteins (Supplementary Fig. S1). This suggests that the cognate wildtype residue may be essential for protein function. In agreement with these residues being important to protein function, the G59D and R145H variants are located close to variants demonstrated to result in a loss-of-function for Single Minded 1 (SIM1; T46R) or NPAS4 (F147S) (Supplementary Fig. S1). Only one in-silico prediction software utilised identified the G59D variant as likely to impact protein function (1/3 damaging), while the E99K and R145H variants were identified as more likely to disrupt protein function (2/3 and 3/3 damaging respectively) (Table 1). Like *NPAS3*, all *NPAS4* single nucleotide variants were identified at a low frequency (< 0.003%) in the “non-neuro” gnomAD population (Table 1). We also selected a nonsense variant in NPAS4 that occurs at position 170 within the protein (170X) and is absent from gnomAD. The 170X variant truncates the protein prior to the end of PAS A (Fig. 1), causing the loss of the C-terminal activation domain and is therefore predicted to result in loss of function.

The available patient data indicate that most younger patients with NPAS3 variants (2/2 individuals with G229R and 2/3 individuals with G201R) present with developmental delays, including speech and/or motor delays (Supplementary Table S1), which can be indicative of later intellectual disability^50^. Interestingly, *Npas3*^−/−^ mice also present with altered gait, as well as, impaired fine motor skills and balance^10,13^. One individual identified with a NPAS3 G201R variant did not present with developmental delay, however as low penetrance alleles may exist, the variant was still characterised. Finally, the older individual with the NPAS3 214fs has been diagnosed with ID (Supplementary Table S1). Similarly, the older individuals with NPAS4 E99K and R145H variants have been diagnosed with ID, whereas the younger individuals with the G59D and 170X variants have been diagnosed with developmental delays (Supplementary Table S2). All individuals were heterozygous for the variant allele. It must be noted that the individuals with *NPAS3* and *NPAS4* variants also present with additional, often variable, phenotypic features (detailed in Supplementary Tables S1 and S2), and in some cases other potential causative variants have been proposed (Supplementary Tables S1 and S2).

### Identifying loss-of-function variants

**Figure 2:**
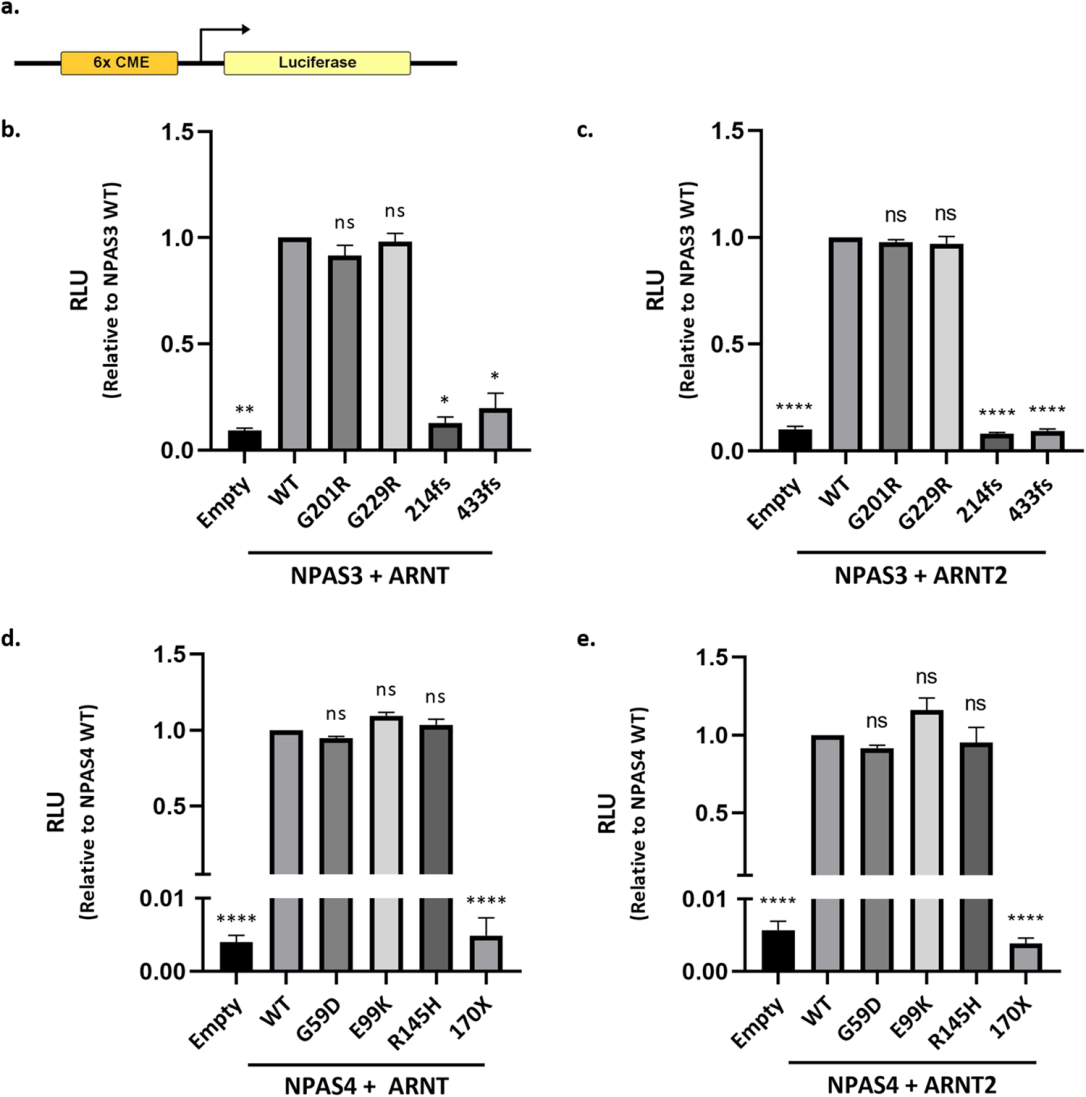
Identification of variants with deficient activity. (**a**) The firefly luciferase reporter gene construct utilised to assess *NPAS3* and *NPAS4* variant activity. The reporter contains an upstream CME site able to be bound by both NPAS3 and NPAS4. All experiments also incorporated renilla luciferase under a constitutive promoter as an internal control. (**b–e**) To test variant activity, HEK293T cells were transiently transfected with the 6 × CME firefly luciferase plasmid, the constitutive renilla luciferase plasmid, and expression plasmids for the *NPAS3/4* variant and either *ARNT* or *ARNT2*. No dimer-specific effects were observed for either NPAS3 or NPAS4. For NPAS3 only the frameshift mutations (214fs and 433fs) had deficient activity (**b**,**c**). For NPAS4 only the nonsense variant (170 ×) had deficient activity (**d**,**e**). All graphs show mean with SEM from at least 3 independent experiments, each performed in triplicate. Significance was calculated by Ordinary One-Way ANOVA with Dunnett’s multiple comparison test on log-transformed raw data (*p < 0.05, **p < 0.01, ****p < 0.0001). *RLU*—Relative Luciferase Units, *WT*—wildtype, *ns*—not significant.

To assess the impact of *NPAS3/4* variants on transcription factor activity we utilised a dual luciferase reporter gene assay where the expression of firefly luciferase was under the control of the Central Midline Enhancer (CME) (Fig. 2a). NPAS4 was previously demonstrated to bind and activate a reporter gene under the control of the CME^39^. NPAS3 was shown to be able to bind to the Hypoxia Response Element (HRE) with the sequence TACGTG^51^, which is identical to the core CME element used for the reporter gene. NPAS3 is known to be a transcriptional activator in HEK293T cells^28^, and in accordance with this we found that NPAS3 increased expression of the reporter gene (Fig. 2). Therefore, we characterised both the NPAS3 and NPAS4 variants using the CME-luciferase reporter gene.

Initially, a *NPAS3* variant that leads to a frameshift near the end of PAS B (c.1298delT; 433fs) (Fig. 1 and Supplementary Fig. S1) was also selected for characterisation, however, it was later determined that this was a low confidence variant (present in 2/20 reads) and as such was not included as a disease variant. As NPAS3 433fs disrupts the NPAS3 protein at an analogous position to the previously identified “dimerisation hotspot” within SIM1^43^ (Supplementary Fig. S1), we chose to investigate whether it was sufficient to inhibit NPAS3 dimerisation and transcriptional activity. Therefore, despite the NPAS3 433fs variant not being conclusively identified in a patient, the analysis of this mutation will help determine the likely functional consequences of future NPAS3 variants in this hotspot region that are identified in patients.

It is thought that the neuronal class I bHLH-PAS factors preferentially dimerise with the neuronally enriched class II factor ARNT2^52–54^, however, recent evidence of an in-vivo interaction between NPAS4 and ARNT has been published^55^. Accordingly, all variants were characterised with both ARNT and ARNT2 partner proteins, which allowed us to detect any possible dimer-specific effects.

The NPAS3:ARNT and NPAS3:ARNT2 heterodimers were able to activate the reporter gene and no heterodimer-specific effects were observed (Fig. 2b,c). Of the NPAS3 variants, G201R and G229R had similar reporter activity to the wildtype protein, whereas both the 214fs (7- to 12-fold decrease relative to wildtype; ARNT p < 0.05, ARNT2 p < 0.0001) and 433fs (5- to 10-fold decrease relative to wildtype; ARNT p < 0.05, ARNT2 p < 0.0001) variants had a near complete loss in reporter gene activity (Fig. 2b,c). Similarly, NPAS4 was able to increase reporter gene activity when partnered with either ARNT or ARNT2, and no heterodimer-specific effects were observed (Fig. 2d,e). The NPAS4 variants G59D, E99K and R145H did not differ from wildtype activity, whilst the truncating 170X variant had close to background levels of reporter gene activity (> 200-fold decrease relative to wildtype; ARNT and ARNT2 P < 0.0001) (Fig. 2d,e).

### Mechanism for variant loss-of-function

**Figure 3:**
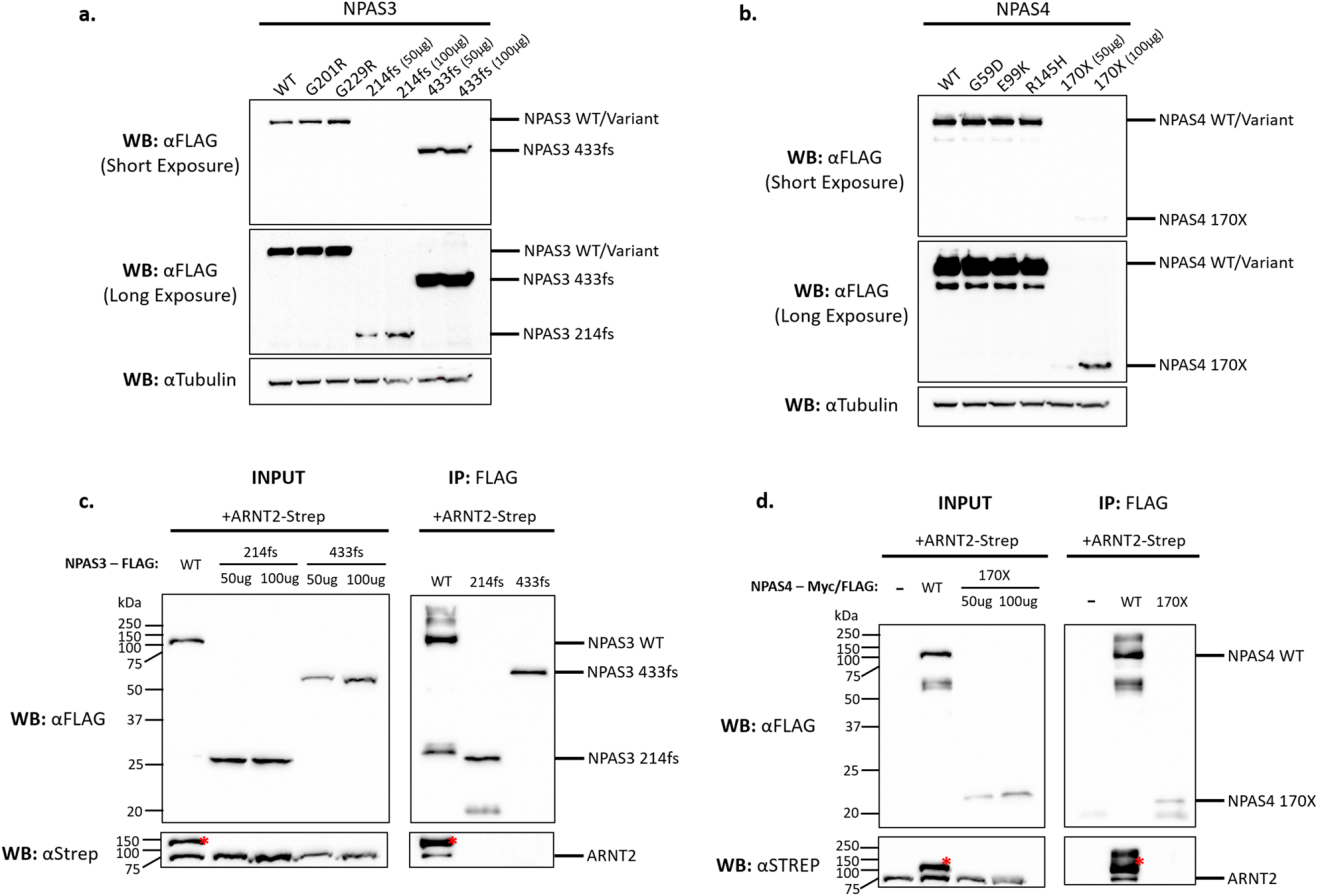
Mechanism for variant loss of function. (**a**,**b**) HEK293T cells were transiently transfected with the *NPAS3* or *NPAS4* variants, and expression was detected by western blot. The NPAS3 214fs and NPAS4 170X variants were expressed at a lower level than the wildtype protein. For clarity of protein expression, both short and long exposures of the same western bot have been presented. All replicates are included in Supplementary Fig. S2, S3. (**c**,**d**) HEK293T cells were transiently transfected with a variant of either NPAS3-FLAG or NPAS4-Myc/FLAG and ARNT2-Strep. 500 μg of whole cell lysate was then subjected to FLAG immunoprecipitation. Unless otherwise stated the input sample included 50 μg of whole cell lysate. (**c**) For NPAS3 all constructs were expressed in the inputs as expected, however, only the wildtype NPAS3 was able to co-immunoprecipitate ARNT2 in the IP sample. (**d**) For NPAS4 all constructs were expressed in the inputs as expected, however, only the wildtype NPAS4 was able to co-immunoprecipitate ARNT2 in the IP sample. Red asterisks represent bleed-through bands from wildtype NPAS3/4, as the blots were probed sequentially with anti-FLAG and then anti-Strep. Blots have not been merged with marker images and are representative of at least 3 independent experiments. For all experiments, full blots merged with markers are available as Supplementary Fig. S2–S4. *WT*—wildtype, *IP*—immunoprecipitation, *WB*—western blot.

To determine whether protein expression was influencing the reporter activity of the variants western blots were performed on whole cell extracts from transfected HEK293T cells. For both NPAS3 and NPAS4, the missense variants were expressed at a similar level to wildtype (Fig. 3a,b). In addition, the NPAS3 433fs was also expressed at a similar level to wildtype (Fig. 3a). In contrast, the NPAS3 214fs and NPAS4 170X variants were expressed at a much lower level than wildtype (Fig. 3a,b), which may partially explain the observed loss of function in the reporter gene assays.

Given the strong loss of function we observed for NPAS3 214fs and NPAS4 170X there is the possibility of a dominant negative effect in which the truncated protein may sequester ARNT/ARNT2 into non-functional heterodimers. This would reduce the availability of the ARNT/ARNT2 proteins for heterodimerisation with wildtype protein (from the other allele) resulting in lower activity than would be expected of a heterozygote. As both NPAS3 214fs and NPAS4 170X were still expressed and possess an intact bHLH domain, the primary site of dimerisation, they may retain the ability to heterodimerise with partner proteins ARNT or ARNT2. The ability of similar AhR (Aryl-hydrocarbon Receptor) constructs containing the bHLH domain and part of PAS A have previously been shown to interact with ARNT in a dominant negative fashion^48^. In addition, as the NPAS3 433fs variant retained a level of expression similar to WT and the majority of the N-terminal domains it may also heterodimerise with ARNT/ARNT2.

To determine if the truncated NPAS3 and NPAS4 proteins were still able to heterodimerise, co-immunoprecipitation (Co-IP) experiments were performed. No functional difference between ARNT and ARNT2 was observed in our reporter gene assays, therefore only the neuronally enriched ARNT2^52^ was utilised for subsequent experiments. Both the NPAS3 and NPAS4 wildtype proteins were able to co-immunoprecipitate ARNT2 (Fig. 3c,d). Importantly, in the absence of NPAS4, ARNT2 was absent from the Co-IP samples, indicating that ARNT2 did not interact non-specifically with the resin or antibody (Fig. 3d). In contrast, NPAS3 214fs, 433fs and NPAS4 170X were unable to co-immunoprecipitate ARNT2 (Fig. 3c,d). Therefore, the loss-of-function variants were unable to heterodimerise with the essential partner protein ARNT2, which explains the mechanism for the loss of function observed, whilst also ruling out a dominant negative effect.

In summary, two loss-of-function variants NPAS3 214fs and NPAS4 170X, were identified in individuals with either ID or developmental delay. This provides evidence that a heterozygous loss-of-function in *NPAS3/4* may contribute to neurodevelopmental disorders. For both variants, the identified mechanism for the loss of function was an inability to heterodimerise with partner protein ARNT2 and decreased protein expression. Finally, the NPAS3 433fs mutation located near the end of PAS B also resulted in a loss of function due to an inability to heterodimerise with ARNT2.

## Discussion

We identified two loss-of-function variants that disrupt PAS A, NPAS3 214fs and NPAS4 170X, in individuals with ID and speech delay respectively. In addition, the NPAS3 433fs mutation that occurs near the end of PAS B and leads to a loss of function was also characterised. In each case the primary reason for loss of function was an inability of NPAS3/4 to heterodimerise with partner protein ARNT2. This highlights the importance of both PAS A and PAS B for dimerisation in-vivo. Characterisation of these variants will help guide future studies that identify variants which truncate either NPAS3 or NPAS4.

NPAS3 214fs is the third variant identified in an individual with developmental delay or ID that truncates or deletes part of *NPAS3*^20,27^, however, it is the first such variant that has been characterised and shown to cause a loss of function. This adds to the growing literature that implicate *NPAS3* variants in neurodevelopmental disorders. Our results can also be used to inform the likely functional consequences of other *NPAS3/4* variants identified in individuals with SZ or neurodevelopmental disorders. For example, our data suggest that the SZ-associated *NPAS3* variant that causes a frameshift just before PAS A^22^ would lead to a complete loss of function due to an inability to heterodimerise with ARNT2. Interestingly, individuals with heterozygous loss-of-function for NPAS3 present with a spectrum of phenotypes, including SZ, ID and SZ with ID. This is in agreement with the observation that SZ is often co-morbid with ID^3^ and that cognitive impairments can be present before SZ is diagnosed^2^.

The presence of loss-of-function *NPAS3* variants in individuals with ID is supported by recent work that screened for genes indirectly and directly regulated by NPAS3^17^. The authors found that several NPAS3 target genes were associated with either ID or SZ in the literature. Of interest are two direct NPAS3 target genes that are normally upregulated by NPAS3, Fragile X Mental Retardation Protein translational regulator 1 (*FMR1*) and Ubiquitin-Protein Ligase E3A (*UBE3A*). In the absence of NPAS3, both targets have decreased expression, which results in downstream pathways of FMR1 and UBE3A being dysregulated^17^. Dysfunction of both FMR1 and UBE3A are known to cause syndromic forms of ID, fragile X syndrome (OMIM: 300624) and Angelman syndrome (OMIM: 105830) respectively. In isolation, a heterozygous loss-of-function for NPAS3 does not appear to be sufficient to cause similar syndromic forms of ID; for example, the individual with atypical Sotos syndrome only presents with low IQ/developmental delay^27^. However, it is possible that in other individuals with a suspected genetic disorder, a loss-of-function for NPAS3 may contribute to a syndromic form of ID.

The NPAS4 170X variant is the first identified loss-of-function allele for *NPAS4* in an individual with developmental delay. There is evidence to suggest that developmental delays can be the precursor of ID^50^, however, there is also a significant environmental influence on neurodevelopmental disorders^56,57^. More specifically for NPAS4, it has been demonstrated that *Npas4*^+/−^ mice exposed to juvenile stress present with cognitive deficits in adulthood when compared to heterozygotes that were stressed in adulthood or not at all^58^. Therefore, environmental factors will have a significant influence on whether the individual with the NPAS4 170X variant, which based on our assays is analogous to being of *NPAS4*^+/−^ genotype, will develop ID. The translation of loss-of-function NPAS4 variants to human phenotypes is further complicated by the observation that in mouse models there appears to be compensatory pathways for NPAS4 target genes^33^ and regulation of synapses^30^. As when compared to an ex-vivo knockout of *Npas4*, the phenotypes of the germline knockouts are attenuated or indistinguishable from wildtype. Therefore, the identification of causative *NPAS4* neurodevelopmental disorder alleles may be complicated by variable penetrance.

Interestingly, the NPAS3 433fs mutation which retains a wildtype sequence until near the end of PAS B was unable to bind ARNT2. The NPAS3/ARNT crystal structure of the highly homologous mouse proteins^51^ utilises truncated proteins such that NPAS3 terminates at the equivalent of human amino acid 448. As this NPAS3 construct can heterodimerise with ARNT, it may indicate that the 15 amino acids that are altered in NPAS3 433fs make essential inter- and intra-molecular contacts, such that the absence of these residues may destabilise PAS B and inhibit heterodimer formation. Alternatively, the 78 amino acids introduced by the frameshift may sterically hinder the ability of NPAS3 433fs to form a dimer with ARNT2. The inability of NPAS3 433fs to heterodimerise with ARNT2 strengthens the previous observation for SIM1^43^ of a heterodimerisation hotspot within this region (Fig. S1). In contrast, NPAS3 214fs disrupts the PAS A/PAS A interface between NPAS3 and ARNT observed in the crystal structure and mutations at this interface have previously been shown to be important for heterodimerisation (Interface 2 in^51^). It is therefore not surprising that NPAS3 214fs was unable to heterodimerise with ARNT2. As no crystal structure exists for NPAS4, similar conclusions about NPAS4 170X are not possible. Overall, the results presented here will help guide future studies which aim to determine NPAS3/4 variant severity, as our work demonstrates that a dominant negative effect is highly unlikely.

We cannot eliminate the possibility that the other variants may have deficits in activity that our reporter system was unable to detect. For example, the variants that were not identified to have a loss of function may have important roles in interacting with context-specific partner proteins. It was recently shown that another bHLH-PAS protein SIM2 was able to interact with MAGED1 (Melanoma-associated antigen D1) through both its PAS A and PAS B domains, resulting in increased SIM2 transcriptional activity^59^. This increase in transcriptional activity by MAGED1 was also shared with NPAS4 (NPAS3 was not tested)^59^. As several variants characterised in this study were in PAS A, they may influence these types of interactions in-vivo. There is some evidence to suggest context-specific partners for NPAS4, as it is known to have different roles in excitatory and inhibitory neurons^31^. The cell environment may also play a role in our ability to detect loss-of-function variants. As NPAS4 is normally active in neurons after depolarisation^30,33^, our assays performed in HEK293T cells under normal cell culture conditions may lack key environmental factors required to observe a loss of function. However, it has previously been shown that *NPAS4* variant activity was not significantly different between HEK293T cells and the neuronal N2A cell line^39^. Furthermore, HEK293T cells were utilised to characterise human variants of *SIM1* that contributed to obesity^60^. Therefore, HEK293T cells are well established for the study of the intrinsic properties of bHLH-PAS transcription factors.

Finally, as both the NPAS3 214fs and NPAS4 170X variants lead to the introduction of premature termination codons (PTCs) there is the possibility that the transcripts will be targeted for nonsense-mediated decay (NMD) and the truncated protein will not be synthesised. However, it has been shown that there is significant inter-person variability in NMD efficiency that can influence disease pathogenicity^61^. This is confounded by the report that the majority of predicted NMD targets are not degraded^62^. Given that there is a strong possibility that in some individuals the mRNA will survive, and the truncated protein will be produced, these variants should still be considered.

To date, the involvement of NPAS3 and NPAS4 in neurodevelopmental disorders has not been extensively explored. Here we have identified loss-of-function variants in both *NPAS3* and *NPAS4* in individuals with ID and developmental delay respectively, further establishing them as candidate neurodevelopmental disorder genes. In the future, large cohort studies looking for additional variants and further phenotypic characterisation will be required. As both loss-of-function variants described here are absent from gnomAD, targeted sequencing of the genes may be required. This is analogous to the approach used previously to identify rare, differentially penetrant variants in another bHLH-PAS factor *SIM1* that were associated with obesity^60,63^. The identification of additional *NPAS3* and *NPAS4* variants will further our understanding of rare variants that may contribute to SZ or neurodevelopmental disorders which could then assist in patient diagnosis.

## Materials and methods

### Exome sequencing database

Clinical exome sequencing performed at Baylor Genetics has previously been reported^64^. The database also included de-identified patient phenotype information.

### Plasmid construction

pEF-BOS NPAS4-Myc/FLAG (ref seq: NM_178864.4) has been previously described^39^. hNPAS3 (refseq: NM_001164749.2) was ordered from Genscript (USA) and was expressed from the EF-BOS vector. All NPAS3 and NPAS4 mutants were cloned by overlap extension PCR as previously described^39^. Puro6-ARNT2-Strep (refseq: NM_014862.4) was cloned by digestion of Puro6-ARNT2 with EcoRV and ligation of annealed Strep tag primers (Upper: 5′ GGAGCGCTTGGAGCCACCCGCAGT TCGAAAAAGGTGGAGGTTCTGGCGGTGGATCGGGAGGTTCAGCGTGGAGCCACCCGCAGTTCGAGAAAGGTtag 3′; Lower: 5′ ctaACCTTTCTCGAACTGCGGGTGGCTCCACGCTGAACCTCCCGATCCACCGCCAGAACCTCC ACCTTTTTCGAACTGCGGGTGGCTCCAAGCGCTCCC 3′). Puro6-ARNT2-FLAG (refseq: NM_014862.4) was sub-cloned from pEF-IRESneo-ARNT2-3xFLAG^39^. Puro6-His/Myc-ARNT^65^ (refseq: NM_178427.3) and the Puro6 backbone^66^ have both been described previously. pML-6xCME-Luciferase (Firefly) was a gift from Dr J. Pelletier (Department of Biochemistry, McGill University, Montreal, Canada).

### Cell culture and transfections

HEK293T cells (ATCC) were grown in DMEM + HEPES (Gibco) supplemented with 10% FCS [Sigma or Mediatech (Corning)], 1 × glutamax (Gibco), 1 × penicillin and streptomycin (Gibco) at 37 °C in the presence of 5% CO_2_. All transfections were performed 24 h after cells were seeded using PEI (polyethylenimine) (Polysciences, USA). In all experiments the ratio of PEI:DNA was 3 μg:1 μg.

### Dual luciferase assay

HEK293T cells seeded in a 24-well tray were transfected with 200 ng pML-6xCME-Luciferase (Firefly), 0.1 ng pRL-CMV (Promega), 50 ng of pEF-BOS expression vectors for either ARNT2-FLAG or His/Myc-ARNT and 50 ng of either NPAS3-FLAG variants or NPAS4-Myc/FLAG variants. 24 h later the cells were lysed with Passive Lysis Buffer (Promega) and luciferase activity was assayed in 10 μL of lysate using the Dual-Luciferase Reporter Assay System (Promega). Relative Luciferase Units (RLU) were calculated by dividing the firefly luciferase value by the renilla luciferase value. Each sample was assayed in triplicate and all experiments were independently repeated at least 3 times.

### NPAS variant expression levels

HEK293T cells seeded in a 6 cm dish were transfected with 1.5 μg of the NPAS variant and 1.5 μg of Puro6 empty plasmid to increase transfection efficiency. 48hrs later the cells were washed in cold 1 × PBS and lysed in 20 mM HEPES pH 8.0, 420 mM NaCl, 0.5% NP-40, 25% glycerol, 0.2 mM EDTA pH 8.0, 1.5 mM MgCl_2_, 1 × protease inhibitors, 1 mM DTT, quantified by protein assay (Bio-Rad Protein Assay Dye Reagent Concentrate) and utilised in immunoblotting. All expression experiments were independently repeated at least 3 times.

### Immunoblotting

Samples were separated on an SDS-PAGE gel before being transferred to a nitrocellulose membrane by the Trans-Blot Turbo Transfer System (Bio-Rad). Blots were probed with the following primary antibodies, anti-FLAG M2 (Sigma; Cat. No. F1804-1MG), anti-Tubulinα (Bio-Rad; Cat. No. MCA78G) and anti-Strep (IBA; Cat No. 2-1507-001). The secondary antibodies used were, goat anti-mouse HRP (Pierce; Cat. No. 31430) and rabbit anti-rat HRP (Dako; Cat. No. P0450). All blots were developed with Clarity Western ECL Blotting Substrates (Bio-Rad).

### Immunoprecipitation

HEK293T cells were transfected with 1.5 μg of a NPAS3-FLAG variant or NPAS4-Myc/FLAG variant and 1.5 μg of ARNT2-Strep. In controls that lacked an NPAS variant, 1.5 μg of Puro6 backbone was used. 48 h later, cell lysates were taken as previously described and quantified by bradford assay (Bio-Rad Protein Assay Dye Reagent Concentrate). 500 μg of whole cell extract were utilised in the IP with 20 μL BSA blocked anti-FLAG M2 Affinity Gel (Sigma) in the presence of protease inhibitors (Sigma). The whole cell extract and resin were incubated with the resin for 3 h at 4 °C before being washed 4 times with the IP wash buffer (150 mM NaCl, 10 mM Tris/HCL pH 8.0, 0.1% NP-40, 5% glycerol). The bound protein was then eluted in two steps (30 μL then 20 μL) with 3xFLAG peptide (Sigma) at 250 μg/mL in IP wash buffer without NP-40 for 1.5 h at 4 °C. To ensure no resin was present in the elution, the elution was centrifuged, and the supernatant taken and 35 μL of elution was then utilised for immunoblotting. All immunoprecipitations were independently repeated at least 3 times.

### Statistics

All data in graphs were presented as a mean with SEM. Significance was calculated by Ordinary One-Way ANOVA with Dunnett’s multiple comparison test on log-transformed raw data, using the program GraphPad Prism (v8.0.0).

### Schematic diagrams

The schematic of *NPAS3* and *NPAS4* variants analysed (Fig. 1) and the schematic of the reporter gene construct (Fig. 2a) were created using the IBS software^67^.

## Supplementary Information

Supplementary Information.

## Data Availability

All reagents and data are available upon request.
