# Supplementary material for: Molecular characterisation of rare loss-of-function NPAS3 and NPAS4 variants identified in individuals with neurodevelopmental disorders
Source: Sci Rep. 2021 Mar 23;11:6602. doi: 10.1038/s41598-021-86041-4 (PMC7987981; doi:10.1038/s41598-021-86041-4)
Supplement: Supplementary file 1 — Supplementary Information. [file 41598_2021_86041_MOESM1_ESM.pdf]

Title: Molecular Characterisation of Rare Loss-of-Function *NPAS3* and *NPAS4* Variants Identified in Individuals with Neurodevelopmental Disorders

Authors:

Joseph J. Rossi[1] (joseph.rossi@adelaide.edu.au) , Jill A. Rosenfeld[2,3] (mokry@bcm.edu), Katie M. Chan[2] (kmchan@texaschildrens.org), Haley Streff[2] (hxstreff@texaschildrens.org), Victoria Nankivell[1] (Victoria.Nankivell@sahmri.com), Daniel J. Peet[1] (dan.peet@adelaide.edu.au), Murray L. Whitelaw[1] (murray.whitelaw@adelaide.edu.au), David C. Bersten[1]* (David.Bersten@adelaide.edu.au)

1. Department of Molecular and Biomedical Science, University of Adelaide, Australia, 5005
2. Department of Molecular & Human Genetics, Baylor College of Medicine, Houston, TX, 77030
3. Department of Clinical Genomics, Baylor Genetics Laboratory, Houston, TX, 77030

***Corresponding Author**:

David C. Bersten

E: David.Bersten@adelaide.edu.au

```
                                                                                    bHLH --->
sp|Q8IUM7|NPAS4_HUMAN   ---------------------------------------------------MYRSTKGAS     9
sp|Q8IXF0|NPAS3_HUMAN   MAPTKPSFQQDPSRRERITAQHPLPNQSECRKIYRYDGIYCESTYQNLQALRKEKSRDAA     60
sp|P81133|SIM1_HUMAN    ---------------------------------------------------MKEKSKNAA     9
sp|Q14190|SIM2_HUMAN    ---------------------------------------------------MKEKSKNAA     9
                                                                     ..::.*:

                                E19K                       T46R         G59D
sp|Q8IUM7|NPAS4_HUMAN   KARRDQINAEIRNLKELLPLAEADKVRLSYLHIMSLACIYTRKGVFFAGGTP--------     61
sp|Q8IXF0|NPAS3_HUMAN   RSRRGKENFEFYELAKLLPLPAAITSQLDKASIIRLTISYLKMRDFANQG-DPPWNLRME     119
sp|P81133|SIM1_HUMAN    RTRREKENSEFYELAKLLPLPSAITSQLDKASIIRLTTSYLKMRVVFPEGLGEAWGHSSR     69
sp|Q14190|SIM2_HUMAN    KTRREKENGEFYELAKLLPLPSAITSQLDKASIIRLTTSYLKMRAVFPEGLGDAWGQPSR     69
                        ::** : * *: :* :****  * . :*.   *: *:  * :   .   *
                                                  PAS A --->                  E99K
sp|Q8IUM7|NPAS4_HUMAN   -------------------LAGPTGLLSAQELEDIVAALPGFLLVFTAEGKLLYLSESVS     102
sp|Q8IXF0|NPAS3_HUMAN   GPPPNTSVKVIGAQRRRSPSALAIEVFEAHLGSHILQSLDGFVFALNQEGKFLYISETVS     179
sp|P81133|SIM1_HUMAN    --------------------TSPLDNVGRELGSHLLQTLDGFIFVVAPDGKIMYISETAS     109
sp|Q14190|SIM2_HUMAN    --------------------AGPLDGVAKELGSHLLQTLDGFVFVVASDGKIMYISETAS     109
                                            :     .  .  ..:: :* **::..  :**::*:**:.*
                                              G201R        L214           G229R
sp|Q8IUM7|NPAS4_HUMAN   EHLGHSMVDLVAQGDSIYDIIDPADHLTVRQQLTLPSALDTD------------------     144
sp|Q8IXF0|NPAS3_HUMAN   IYLGLSQVEL--TGSSVFDYVHPGDHVEMAEQLGMKLPPGRGLLSQGTAEDGASSASSSS     237
sp|P81133|SIM1_HUMAN    VHLGLSQVEL--TGNSIYEYIHPADHDEMTAVLTAHQPYH--------------------     147
sp|Q14190|SIM2_HUMAN    VHLGLSQVEL--TGNSIYEYIHPSDHDEMTAVLTAHQPLH--------------------     147
                         :** * *:*   *.*::: :.*.**  :   *
                                                F147S
                                              R145H        R171H          R170X
sp|Q8IUM7|NPAS4_HUMAN   -----------------------RLFRCRFNT---SKSLRRQSAGNKLVLIRGRFHAHPP     178
sp|Q8IXF0|NPAS3_HUMAN   QSETPEPVESTSPSLLTTDNTLERSFFIRMKSTLTKRGVHIKSSGYKVIHITGRLRLRVS     297
sp|P81133|SIM1_HUMAN    -------------SHFVQEYEIERSFFLRMKCVLAKRNAGLTCGGYKVIHCSGYLKIRQY     194
sp|Q14190|SIM2_HUMAN    -------------HHLLQEYEIERSFFLRMKCVLAKRNAGLTCSGYKVIHCSGYLKIRQY     194
                                               * *  *::    .:.    ..* *::   * :: :

                                                        PAS B --->
sp|Q8IUM7|NPAS4_HUMAN   GAYWAGNPVFTAFCA---PLEPRPRPGPGPGPGPASLFLAMFQSRHAKDLALLDISESVL     235
sp|Q8IXF0|NPAS3_HUMAN   ---LSHGR--T-VPSQIMGLVVVAHALPPPTINEVRIDCHMFVTRVNMDLNIIYCENRIS     351
sp|P81133|SIM1_HUMAN    ---SLDMSPFD-GCYQNVGLVAVGHSLPPSAVTEIKLHSNMFMFRASLDMKLIFLDSRVA     250
sp|Q14190|SIM2_HUMAN    ---MLDMSLYD-SCYQIVGLVAVGQSLPPSAITEIKLYSNMFMFRASLDLKLIFLDSRVT     250
                                           *    :  *        :   **  *   *: ::  .. :
                                                                      R296G
                                                                  T292A
sp|Q8IUM7|NPAS4_HUMAN   IYLGFERSELLCKSWYGLLHPEDLAHASAQHYRLLAESGDIQAEMVVRLQAKTGGWAWIY     295
sp|Q8IXF0|NPAS3_HUMAN   DYMDLTPVDIVGKRCYHFIHAEDVEGIRHSHLDLLNKGQC--VTKYYRWMQKNGGYIWIQ     409
sp|P81133|SIM1_HUMAN    ELTGYEPQDLIEKTLYHHVHGCDTFHLRCAHHLLLVKGQV--TTKYYRFLAKHGGWVWVQ     308
sp|Q14190|SIM2_HUMAN    EVTGYEPQDLIEKTLYHHVHGCDVFHLRYAHHLLLVKGQV--TTKYYRLLSKRGGWVWVQ     308
                           .    :::  *  *  :*  *       *  ** :.    .    *   * **: *:
                        S309G        H323Y    L433
sp|Q8IUM7|NPAS4_HUMAN   CLLYSE-----GPEGPITANNYPISDMEAWS     321
sp|Q8IXF0|NPAS3_HUMAN   SSATIAINAKNANEKNIIWVNYLLSNPEYKD     440
sp|P81133|SIM1_HUMAN    SYATIVHNSRSSRPHCIVSVNYVLTDTEYKG     339
sp|Q14190|SIM2_HUMAN    SYATVVHNSRSSRPHCIVSVNYVLTEIEYKE     339
                        .          .    *   ** ::: *
```

Figure S1: Alignment of Neuronal bHLH-PAS factors

The N-terminal amino acid sequences of neuronal bHLH-PAS factors, NPAS3, NPAS4, SIM1 and SIM2, were aligned using the CLUSTAL O (1.2.4) multiple sequence alignment tool. Orange sequence indicates the bHLH domain, blue sequence indicates PAS A and green sequence indicates PAS B. Domain boundaries were determined based on alignments performed for the NPAS1/NPAS3 crystal structure [1]. Relevant loss of function variants previously identified for NPAS4[2], SIM1[3], SIM2 (Whitelaw, unpublished), in addition to all missense/nonsense variants characterised in this study are highlighted in red. Finally, the start location of the two frameshift mutations are also indicated.

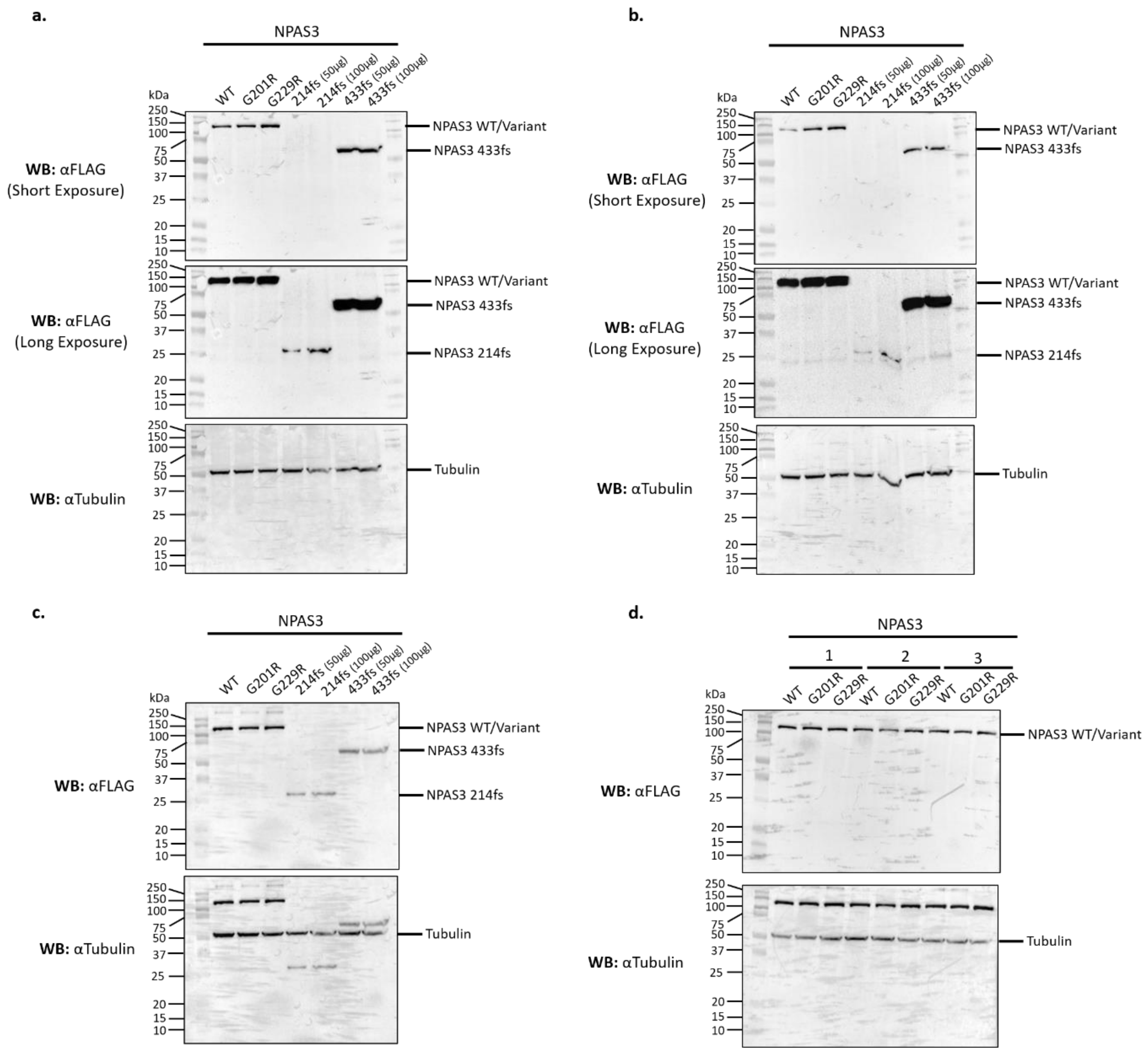

Figure S2: *NPAS3* Variant Expression – Full Blots and Replicates

a.-c. HEK293T cells were transiently transfected with *NPAS3* variants, and expression was detected by western blot. The results of 3 independent experiments are shown. **d.** Due to variability observed in NPAS3 WT expression across **a.-c.** three independent sets of lysates (1-3), were run simultaneously on a single blot. Of note, replicate 1 utilises the same lysates as **c.** and replicates 2 and 3 utilise new lysates. WT= wildtype; WB = western blot.

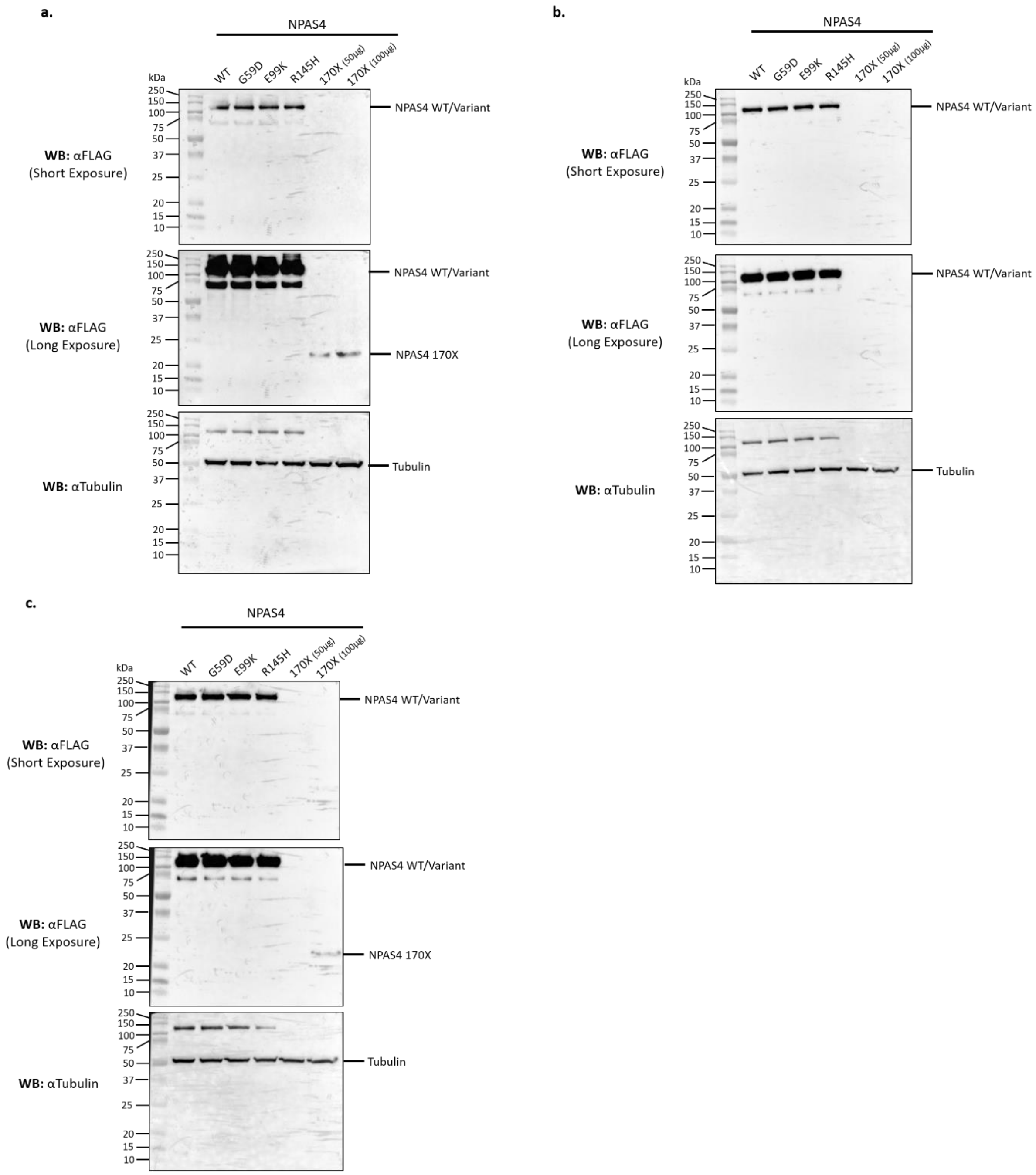

Figure S3: *NPAS4* Variant Expression – Full Blots and Replicates

a.-c. HEK293T cells were transiently transfected with *NPAS4* variants, and expression was detected by western blot. The results of 3 independent experiments are shown. WT= wildtype; WB = western blot.

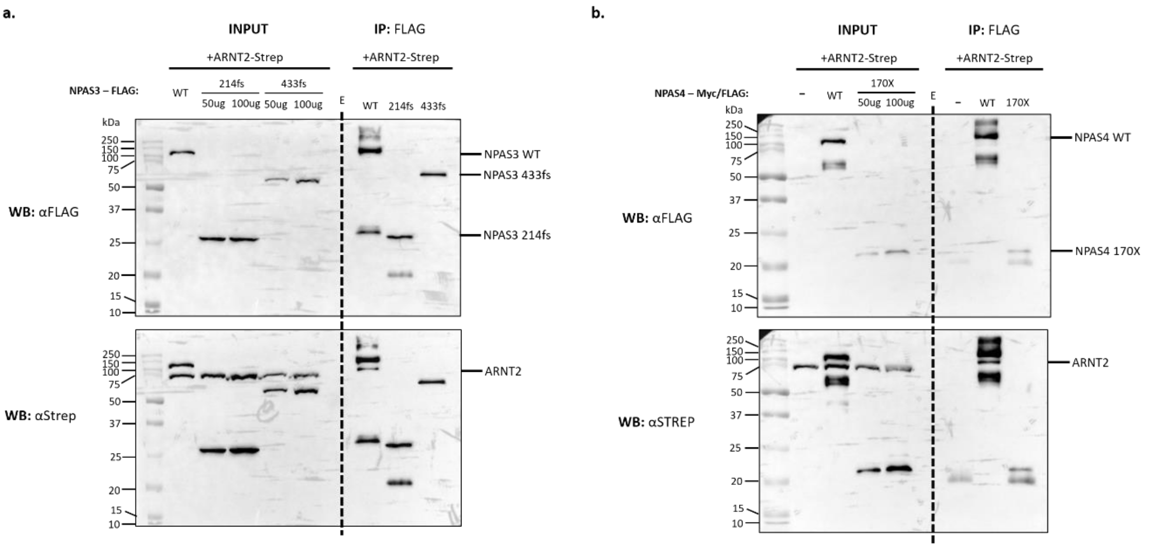

Figure S4: *NPAS3* and *NPAS4* Variant Co-immunoprecipitation of ARNT2 – Full Blots

a.-b. HEK293T cells were transiently transfected with a variant of either NPAS3-FLAG or NPAS4-Myc/FLAG and ARNT2-Strep. 500µg of whole cell lysate was then subjected to FLAG immunoprecipitation. Unless otherwise stated the input sample included 50µg of whole cell lysate. WT= wildtype; WB = western blot; IP = immunoprecipitation; E = lane left intentionally empty.

Table S1: Patient Information for *NPAS3* Variants

Gene	Age [Years] (Sex)	AA Change (Nt Change)	Zygosity	Reads (Var/ Ref†)	Patient Phenotype at Time of Sequencing	Other Mutations/Notes
*NPAS3**	4.3 (M)	p.G201R (c.601G>A)	Het	22/44	Swallowing issues in infancy, motor delays, difficulty walking, frequent falls, ophthalmoplegia, diffuse hypotonia, hyporeflexia, decreased muscle strength and overweight.	A heterozygous c.9847C>T (p.R3283X) variant and a heterozygous c.1250T>C (p.L417P) variant in the *RYR1* gene (refseq: NM_000540.3) were detected in this individual. Mutations in *RYR1* are the cause of multiminicore disease with external ophthalmoplegia (OMIM:255320). Family history is positive for a sister with ophthalmoplegia and the paternal grandmother with walking difficulties.
	0.048 (M)	p.G201R (c.601G>A)	Het	62/143	Congenital heart disease which includes transposition of the great arteries, coarctation of the aorta, and ventricular septal defect (VSD). Additionally, a history of intrauterine growth restriction and extremely low weight.	In a more recent assessment at an age of 3.75 years the individual did not have any developmental issues.
	2.3 (F)	p.G201R (c.601G>A)	Het	58/144	Delayed motor milestones, hypotonia, hypertonia/spasticity, seizure disorder, abnormal movements and microcephaly.	A homozygous c.79T>C (p.Y27H) variant in the *GAMT* gene (refseq: NM_000156.6) was detected in this individual. Homozygous mutations in *GAMT* are known to cause Cerebral Creatine Deficiency Syndrome 2 (OMIM: 612736).
	1.4 (F)	p.G229R (c.685G>A)	Het	25/54	Microcephaly, hypotonic cerebral palsy, profound intellectual disability, esotropia, delayed visual maturation, small optic nerves, nystagmus, mild distal lower extremity hyperreflexia, global developmental delay, and dysmorphic features.	A *de novo* novel heterozygous c.997delG (p.A333fs) variant in the *FOXG1* gene (refseq: NM_005249.5). Mutations in *FOXG1* can cause Congenital Rett Syndrome (OMIM: 613454). Family history is significant for both parents with delays and father with cardiomyopathy.
	2.1 (F)	p.G229R (c.685G>A)	Het	30/70	Intrauterine growth restriction, delayed motor milestones, delayed speech, hypotonia, dysmorphic features, hyperextensibility, short stature, microcephaly, eye anomalies (esotropia), bicuspid aortic valve and partial fusion of aortic leaflets, hemangioma over glabella, congenital hypothyroidism and stereotyped flapping movements of her arms.	A *de novo* heterozygous c.410_411del (p.137fs) variant in the *H3F3B* gene was detected in this individual. Defects in *H3F3B* are not currently associated with a disease in humans.
	16.3 (M)	p.L214fs (c.641dupT)	Het	49/78	Common variable immune deficiency, Evans syndrome, growth hormone deficiency, demyelination findings consistent with multiple sclerosis, eczema, hepatosplenomegaly, speech delay, intellectual disability, short stature, dysmorphic features, failure to thrive, complex dental caries resulting in the removal of all teeth, viral myocarditis, cardiomyopathy, eneuresis, neutropenia, thrombocytopenia, and intermittent headaches.	

* *NPAS3* refseq: NM_001164749.2 †Variant Reads/Reference Sequence Reads

Table S2: Patient Information for *NPAS4* Variants

Gene	Age [Years] (Sex)	AA Change (Nt Change)	Zygosity	Reads (Var/ Ref†)	Patient Phenotype at Time of Sequencing	Other Mutations/Notes
*NPAS4**	1.3 (M)	p.G59D (c.176G>A)	Het	16/30	Profound speech and motor delay, congenital hydrocephalus and congenital brain malformation (Chiari I malformation, absent corpus callosum and possible aqueductal stenosis), oculomotor apraxia, mixed tone abnormalities in the muscle, and poor weight gain.	In a more recent assessment at an age of 3.5 years, was non-verbal and had persistent, profound motor delays and an absent septum pellucidum.
	10.7 (M)	p.E99K (c.295G>A)	Het	21/49	Intrauterine growth restriction with motor delay, speech delay, intellectual disability, hypotonia, seizure, short stature, failure to thrive, and possible developmental regression.	A *de novo* heterozygous c.239T>C (p.I80T) variant in the *GNB1* gene (ref seq: NM_002074.5). Defects in *GNB1* are known to cause intellectual disability (OMIM: 616973).
	23.1 (F)	p.R145H (c.434G>A)	Het	33/63	Seizures, intellectual disability, hypotonia, mild dysmorphisms, and sensorineural hearing loss.	
	2.5 (F)	p.R170X (c.508C>T)	Het	24/51	Speech delay, hypotonia, dysmorphic features (hypertelorism, fetal finger pads, flattened nasal bridge and broad mouth), and hyperextensibility.	Family history indicates an older brother with delayed speech, hypotonia, and hyperextensibility; he does not carry the variant. The mother also has hyperextensible elbows and fingers.

* *NPAS4* refseq: NM_17886 †Variant Reads/Reference Sequence Reads

References

1 Wu, D., Su, X., Potluri, N., Kim, Y. & Rastinejad, F. NPAS1-ARNT and NPAS3-ARNT crystal structures implicate the bHLH-PAS family as multi-ligand binding transcription factors. *Elife* **5**, doi:10.7554/eLife.18790 (2016).

2 Bersten, D. C., Bruning, J. B., Peet, D. J. & Whitelaw, M. L. Human variants in the neuronal basic helix-loop-helix/Per-Arnt-Sim (bHLH/PAS) transcription factor complex NPAS4/ARNT2 disrupt function. *PLoS One* **9**, e85768, doi:10.1371/journal.pone.0085768 (2014).

3 Sullivan, A. E. *et al.* Characterization of human variants in obesity-related SIM1 protein identifies a hot-spot for dimerization with the partner protein ARNT2. *Biochemical Journal* **461**, 403-412, doi:10.1042/BJ20131618 (2014).
